# Relationship between metacognitive awareness and motivation to learn in medical students

**DOI:** 10.1186/s12909-020-02318-8

**Published:** 2020-10-30

**Authors:** Marina Alves Martins Siqueira, Johnatan Padovez Gonçalves, Vitor Silva Mendonça, Renata Kobayasi, Fernanda Magalhães Arantes-Costa, Patricia Zen Tempski, Mílton de Arruda Martins

**Affiliations:** 1 Center of the Development of Medical Education (CEDEM), School of Medicine, University of Sao Paulo, Sao Paulo, SP Brazil; 2 Harvard T.H. Chan School of Public Health, Boston, MA USA; 3 Laboratorio de Terapeutica Experimental (LIM20), Hospital das Clinicas HCFMUSP, Faculdade de Medicina, Universidade de Sao Paulo, Sao Paulo, SP Brazil; 4 Undergraduate Office of School of Medicine, University of Sao Paulo, Sao Paulo, SP Brazil; 5 Department of Internal Medicine, School of Medicine, University of Sao Paulo, Av Dr Arnaldo, 455 Sala 1210, Sao Paulo, SP CEP 01246-903 Brazil

**Keywords:** Metacognition, Motivation, Medical students, Medical education, Learning, Self-regulation

## Abstract

**Background:**

In self-regulated learning, learning is defined as metacognitively guided, intrinsically motivated and strategic. In the context of medical education, the development of self-regulated learning can be associated with better academic and clinical performance. Hence, this report focuses on demonstrating the association between metacognitive awareness and motivation to learn among medical students in the clinical sciences portion of their education (3rd and 4th years of the medical programme) and characterizing medical students’ motivational factors.

**Methods:**

We performed a cross-sectional study with a qualitative and quantitative approach involving medical students from the University of Sao Paulo (USP) in Brazil. We have selected validated self-report questionnaires for the evaluation of metacognition (the Schraw and Dennison Metacognitive Awareness Inventory - MAI) and motivation to learn (the Baranik, Barron and Finney Achievement Goals for a Work Domain - AGWD). MAI has two domains: knowledge about cognition and regulation of cognition. AGWD divides achievement goals into mastery approach, mastery avoidance, performance approach and performance avoidance goal orientations. We also performed a qualitative analysis based on an open-ended question: “What motivates me the most in medical training?”

**Results:**

One hundred eighty-five students completed the questionnaires: 103 (55.67%) were men, 110 (59.45%) were in their fourth year of the medical programme, and 152 (82.16%) were up to 24 years old. Only the knowledge about cognition domain of MAI was significantly associated with motivation to learn. We found that higher scores on the knowledge about cognition domain of MAI was associated with the mastery approach goal orientation (*p* = 0.003, median 0.71, IQR 0.23) and that lower scores on this same domain was associated with a mastery avoidance goal orientation (*p* = 0.034, median 0.65, IQR 0.14). The open-ended question showed that altruism, personal satisfaction, financial feedback, personal and supportive networks and graduating were motivational factors.

**Conclusions:**

Metacognitive awareness and motivation to learn are closely related. This association may represent a potential target for the educational process, as deans and faculty can adopt strategies focused on promoting self-regulated learning concerning students’ motivational factors. This could enhance academic outcomes and promote more enjoyable learning.

## Background

Self-regulated learning (SRL) theory defines learning as a metacognitively guided process [1–3]. SRL suggests that integration among metacognition, strategic learning and motivation is useful for explaining whether some students engage purposefully in learning processes and goal-directed activities [4]. A self-regulated student has cyclical control of her or his learning process. The cycle starts with motivation and previous preparation for a task, such as reading an article about a theme. Then, during learning, the student adjusts or adapts behaviours through learning strategies such as rereading, developing concept maps, creating summaries, completing quizzes, and changing the environment. Finally, the student engages in self-reflection, in which she or he will evaluate her or his own results and modify or adapt goals or strategies for future tasks, which characterizes metacognition awareness [5].

SRL theory incorporates cognitive, affective and social factors, representing an inclusive perspective of students’ learning and their motivations [6]. In the context of medical education, SRL theory can be represented by students who are active participants in their learning and are guided through key processes as strategies to attain goals, adapt behaviours and optimize learning and performance [5, 7]. It was demonstrated that higher levels of SRL are associated with higher academic achievement, more success in learning clinical skills and better mental health outcomes [8]. However, few studies have focused on the relationship among the components of SRL in medical students [9].

Metacognition is the awareness one has about her or his knowledge and the regulation of learning processes to meet the demands of particular tasks [10, 11]. Students who develop metacognitive strategies can plan, monitor and regulate their cognition process. Thus, more efficient study plans, responsibilities and deep learning should be developed [9]. Recent studies have also shown that metacognitive practices result in better patient care [12], clinical reasoning [13], clinical decision making [14], and a continuous process of lifelong learning, which is essential in medical practice [11, 15–17].

According to Brown’s framework, which addresses metacognition within the context of academic learning settings [18], metacognition can be divided into two broad components: knowledge about cognition and regulation of cognition [19]. Knowledge about cognition relates to an awareness about one’s strengths and weaknesses. It is shown by a better self-reflection process [5], the recognition of knowledge and the ability to recognize how, when and why to use learning procedures [5, 20]. Regulation of cognition corresponds to a final evaluation and modification of learning strategies for future learning and includes five skills: planning, implementing, monitoring, debugging and evaluating strategies. The two components are correlated but not compensatory. This means that each one makes a unique contribution to cognitive performance. Students with higher knowledge about cognition have higher test performance, for example [20]. Students with low regulation have the most challenging experiences with planning, organization and elaboration strategies [21].

Motivation to learn is influenced by one’s beliefs in the importance of a particular subject and how it will help them cope with a new circumstance or solve real problems. In addition, the content must dialogue with their previous knowledge to enable comparison and reflection about the new data [22]. Based on social-cognitive learning theory, individuals are motivated to achieve personal goals through the self-regulation of thoughts, actions and environmental factors [5, 23].

Achievement goal orientation theory is based on a social-cognitive framework [24–26] and proposes a new direction to describe motivation as the reason why an individual actively pursues a task [6]. Goals can be categorized into mastery and performance-oriented goals. Mastery-oriented goals are positively associated with better cognition, motivation, and behaviour [27]. The student has a stronger belief that success follows effort and is more prone to pursue challenging tasks, trust their own abilities and face failure as a positive opportunity to improve outcomes. Students oriented towards performance goals, on the other hand, tend to focus on their ability, evaluate their ability negatively and attribute failure to a lack of ability [28, 29].

The ability to balance mastery and performance goals can provide satisfactory outcomes, since performance goals help students identify strengths and weaknesses. However, it can become problematic when the individual starts avoiding learning opportunities for fear of receiving negative feedback. To distinguish these scenarios, Elliot and McGregor propose the categorization of mastery and performance goals into approach and avoidance dimensions, resulting in a 2 × 2 model [30].

Although there is growing interest in SRL, there are few studies that investigate the relationship between its components, especially through multimethod (quantitative and qualitative) analyses [8, 9]. Most previous studies show significant associations between metacognition and motivation in junior high students and undergraduates from a psychology course [11, 31] using quantitative approaches. This study focuses on demonstrating a possible association between metacognitive awareness and motivation to learn in medical students in the clinical sciences portion of their education.

According to previous studies, medical students’ motivation to learn is influenced by interest in science/medicine, social interests, altruism, flexible work hours, prestige, and financial security [32]. Thus, the present study also performs a qualitative analysis to describe the factors that influence their motivations to learn.

## Methods

We performed a cross-sectional study involving medical students enrolled in the clinical sciences portion of their medical programme at the University of Sao Paulo (USP), Brazil.

### Local structure of the medical programme and participants

In Brazil, a medical degree is obtained in a 6-year undergraduate program, which is traditionally divided into three periods: basic sciences (1st and 2nd years), clinical sciences (3rd and 4th years) and clerkships (5th and 6th years). In the basic sciences period, students are exposed to the fundamentals of biochemistry, cellular biology, physics, anatomy, physiology, and pathophysiology. During the clinical sciences period, students have contact with patients, surgeries and activities that integrate theory and practice. In the clerkship period, students mainly perform workplace training (primary care, ambulatories and hospital settings). In the School of Medicine of the University of Sao Paulo, 175 students start as freshmen every year [33]. All the students enrolled in the 3rd and 4th years of the medical programme (*n* = 360) were invited to participate in the study either at the beginning or at the end of a theoretical class.

### Study design

The study combined a quantitative analysis based on validated self-report questionnaires for the evaluation of metacognition (the Schraw and Dennison Metacognitive Awareness Inventory (MAI) [20]) and motivation to learn (the Baranik, Barron and Finney Achievement Goals for a Work Domain (AGWD) [34–38]) and qualitative analysis based on an open-ended question: “What motivates me the most in medical training?” Data collection was performed from July 2018 to September 2018.

The research ethics committee of the School of Medicine of the University of Sao Paulo approved this study. Participation was voluntary, and we did not offer any compensation or incentives. We guaranteed both confidentiality and anonymity, and participating students completed a consent form.

### Socio-demographic assessment

We have collected data about gender, age and year of medical programme of participants.

### Metacognition assessment

In our study, we choose to analyse metacognition awareness as a manifestation of metacognition [39]. There are other ways to assess metacognition, such as SRL microanalytic assessment questions [4] and the think-aloud (TA) technique [40], and recent studies also point to imaging exams such as electroencephalography (EEG) [41]. However, extensive verbal interviews and the use of medical equipment would not be feasible for our population of interest.

In this sense, we chose to use a self-report statement-based inventory developed by Schraw and Dennison called the Metacognitive Awareness Inventory (MAI). The MAI is widely used in the field of education [42] because of its easy application and reliability [20]. The translated and validated Brazilian Portuguese version consists of 14 items clustered in 2 domains: knowledge about cognition and regulation of cognition [39]. The statements were answered based on a 5-point Likert scale from “never true for me” (1) to “always true for me” (5), and the mean scores were calculated for each domain separately for statistical analysis. To avoid overstimulation of academic abilities, we made only one application as recommended by the instrument developers. The Cronbach’s alpha value for this scale was 0.84, without showing ceiling and floor effects.

### Motivation to learn assessment

The Baranik, Barron and Finney Achievement Goals for a Work Domain (AGWD) is a short-form questionnaire to measure motivation in a labour context [34]. In the clinical sciences period of medical training, practical activities are predominant, supporting the election of this instrument. The translated and validated Brazilian Portuguese version comprises 18 items, each specifically associated with one of 4 achievement goals: mastery approach, mastery avoidance, performance approach, and performance avoidance [43].

- The mastery approach (MAP) is associated with a self-referral improvement pattern. The students’ priority is the development of competencies and skills, deeper learning, satisfaction, stress tolerance and well-being. Students are motivated by academic activities.
- The performance approach (PAP) is associated with intersubjective improvement patterns. Students are focused on recognition from others and are motivated by results, grades, and feedback.
- Performance avoidance (PAV) is associated with a fear of incompetence and the avoidance of failure and negative feedback. There is a predominance of feelings of worry, anxiety and procrastination. Students are less motivated and give up more easily.
- Mastery avoidance (MAV) is associated with a fear of showing weakness and academic difficulties. It is characterized by inadequate coping and learning strategies. Students are motivated by achieving the minimum.

The statements were answered on a 7-point Likert scale from “not at all characteristic” (1) to “very characteristic” (7), and each of the items corresponds to one specific achievement goal. Thus, the definition of one’s goal orientation is based on the achievement goal that shows the highest sum. Correlations between the four goal orientations revealed that they were related, yet independent [34]. Thus, we decided to convert scores into a binary outcome, focusing on the predominant goal orientation for analysis. The Cronbach’s alpha value for this scale was 0.79, without showing ceiling and floor effects.

Finally, we included an open question to assess the students’ motivations: “What motivates me the most in medical training?”

### Statistical analysis

Descriptive statistics were used to analyse demographic data and students’ metacognitive and motivational scores. The Mann-Whitney U test was used to compare measures of the central tendency of scores of *metacognitive awareness* according to gender, year in the medical programme and age group. Fisher’s exact test was used to verify possible correlations between demographic characteristics and goal orientations.

We established the level of statistical significance as 0.05. All statistical analyses were performed using SPSS Statistics for Windows, Version 22.0 (released 2013, IBM Corp, Armonk, NY).

Qualitative analysis was based on the open-ended question attached to questionnaires. Responses were transcribed for analysis and categorized according to traditional methods of content analysis [44]. Two independent researchers started with a free reading of the transcribed text, without the intention of categorization. During the second reading, the researchers proceeded to the categorization of emerging themes and derived issues separately. Finally, each researcher’s products were paired by similarities in meaning and were discussed with the research group. The results were divided into analytical categories, items and examples.

## Results

Of 360 students in the clinical sciences period, 222 were included in the study (61.67%). The main reasons why students refused to participate were a lack of time and absence in class. Therefore, 185 participants completed the questionnaires. Thirty-seven (10.27%) left more than 5% of the items on the MAI or AGWD blank, so they were considered missing data.

Regarding sample characteristics, 103 (55.67%) were men, 110 (59.45%) were in their fourth year of the medical programme, and 152 (82.16%) were up to 24 years old.

Concerning the assessment of metacognition, male students had higher scores on the domain of knowledge about cognition on the Metacognitive Awareness Inventory (*p* = 0.045). There were no significant differences in metacognitive awareness scores between students enrolled in their third or fourth year of the medical programme and age group (Table 1). There were no statistically significant differences in goal orientation between gender, year of medical programme and age group (Table 2).

**Table 1:** Metacognitive awareness scores according to age, gender and year of medical programme

	Metacognitive awareness					
	Knowledge about cognition			Regulation of cognition		
	Median	IQR	p*	Median	IQR	p*
**Gender**						
Male (*n* = 103)	0.66	0.14	0.045	0.66	0.17	0.546
Female (*n* = 82)	0.66	0.17		0.66	0.17	
**Year of medical course**						
3rd (*n* = 75)	0.66	0.2	0.997	0.69	0.2	0.294
4th (*n* = 110)	0.66	0.11		0.66	0.14	
**Age group**						
Up to 24 (*n* = 152)	0.66	0.11	0.661	0.66	0.17	0.397
> 24 (*n* = 33)	0.66	0.17		0.66	0.16	

*Mann-Whitney U-test

**Table 2:** Goal orientation according to age, gender and year of medical programme

	Goal orientation			
	MAP	MAV	PAP	PAV
**Gender**				
Male	17 (74%)	81 (53%)	2 (100%)	0
Female	6 (26%)	72 (47%)	0	1 (100%)
p*	0.06	0.102	0.504**	0.443**
**Year of medical course**				
3rd	11 (48%)	63 (41%)	0	0
4th	12 (52%)	90 (59%)	2 (100%)	1 (100%)
p*	0.477	0.7	0.515**	1**
**Age group**				
Up to 24	21 (91%)	124 (81%)	2 (100%)	1 (100%)
> 24	2 (9%)	29 (19%)	0	0
p*	0.380**	0.386	1**	1**

*Pearson’s chi-square test

**Fisher’s exact test

Relative to their motivation to learn, the majority of students (153 or 83%) presented with a mastery avoidance goal orientation, while 12.5% had a mastery approach orientation, 1% had a performance approach orientation, and 0.5% had a performance avoidance orientation.

Our main finding was that students with a mastery approach goal orientation had higher scores on the domain of knowledge about cognition only (*p* = 0.003, median 0.71, IQR 0.23), while mastery avoidance goal-oriented students had lower scores on this same domain (*p* = 0.034, median 0.65, IQR 0.14). There were no significant differences between the domain of regulation of cognition and goal orientation (Table 3).

**Table 3:** Correlation between metacognition and motivation to learn in medical students

			Mastery-approach			Mastery-avoidance		
			Yes *n* = 23	No *n* = 162	p*	Yes *n* = 153	No *n* = 32	p*
**Metacognitive awareness**	Knowledge about cognition	Median	0.71	0.65	0.003	0.65	0.7	0.034
		IQR	0.23	0.14		0.14	0.19	
	Regulation of cognition	Median	0.68	0.65	0.069	0.65	0.68	0.167
		IQR	0.17	0.17		0.17	0.14	

*Mann-Whitney U-Test

**Fig. 1:**
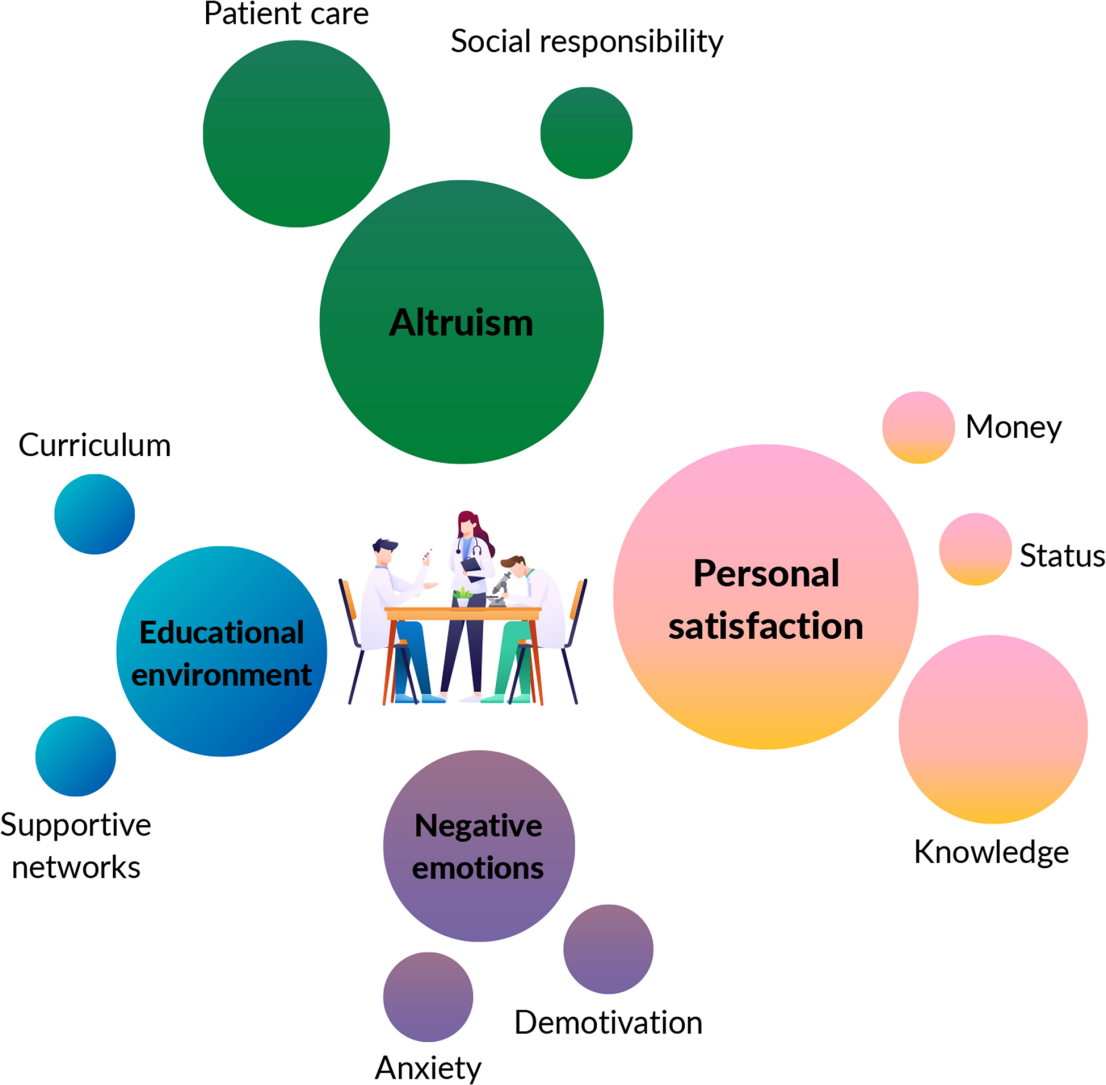
Graphic representation of qualitative data obtained by the open-ended question. The circles are proportional to the number of responses of each category/issue

The responses to the open-ended question “What motivates me the most in medical training?” were organized into 4 categories and divided into 9 issues (Table 4). The majority of responses were included in the personal satisfaction category, followed by altruism, educational environment and negative emotions (Fig. 1).

**Table 4:** Qualitative data produced by the open-ended question “What motivates me the most in medical training?”

Category	Issues	Examples
Personal satisfaction	Knowledge	“I am motivated by the medical course. I really like to study medicine.”
		“It is really exciting to seek new information in order to become the best doctor, in technical and humanistic aspects.”
		“I am very fond of learning and having good medical training.”
	Money	“I feel motivated to keep studying when I think I’m going to be very rich in the future.”
		“The prospect of the financial security offered by a medical career keeps me motivated to go on.”
		“I want to improve mine and my family’s financial conditions by becoming a doctor.”
	Status	“I really look forward to becoming a surgeon.”
		“My main goal is to finish my undergraduate course and enroll in a specialization in psychiatry.”
Altruism	Patient care	“What motivates me the most in medical training is the conviction that in the future, I will participate in patients’ histories and make efforts to make their lives better through the knowledge I have acquired.”
		“I like to promote health through patients’ expectations and understanding of their disease.”
		“I am motivated by the challenge of learning and practising medicine, as long as it involves a high impact on people’s lives.”
		“The patient’s feedback is very important for me to become a good professional.”
	Social responsibility	“I feel happy to be able to experience different scenarios and positively impact people lives.”
Educational environment	Curriculum	“I enjoy activities that involve good case discussions based on patients.”
		“I feel motivated when faculty promote activities in which I can talk to patients.”
	Supportive networks	“My friends are who motivate me the most in academic environments.”
		“I feel motivated to be part of a sports team in the University Athletic Association.”
		“I believe my main motivation is to be enrolled in some extracurricular activities offered by my university.”
Negative emotions	Anxiety	“I just want to finish the undergraduate course as quickly as possible.”
		“I try to keep in mind that the course will end soon.”
	Demotivation	“The course doesn’t motivate me.”

### Personal satisfaction

The majority of responses were included in this category, divided into issues of knowledge acquisition, financial feedback, and social status. Most students reported that learning was their main motivation. Other responses referred to learning how to become a good doctor in terms of building a career and improving socioeconomic status.

*“I am motivated by the medical course. I really like to study medicine.”*

*“It is really exciting to seek new information in order to become the best doctor, in technical and humanistic aspects.”*

*“I am very fond of learning and having good medical training.”*

### Altruism

Students’ sense of altruism referred mostly to individualized patient care as motivation to continue studying. They seemed to appreciate the opportunity to demonstrate their social responsibility and take care of their patients, improving health outcomes and population quality of life. We identify this trait in the following examples:

*“What motivates me the most in medical training is the conviction that in the future, I will participate in patients’ histories and make efforts to make their lives better through the knowledge I acquire.”*

*“I like to promote health through patient’s expectations and understanding of their disease.”*

*“I am motivated by the challenge of learning and practising medicine, as long as it involves a high impact on people’s lives.”*

*“The patient’s feedback is very important for me to become a good professional.”*

*“Studying medicine is a great opportunity to act in favour of society.”*

### Medical programme

Another sample of responses converged to practical curricular activities, participation in student organizations and social relationships as components of medical students’ experience that motivated them during medical training.

*“I enjoy activities that involve good case discussions based on patients.”*

*“I feel motivated when faculty promote activities in which I can talk to patients.”*

*“My friends are who motivate me the most in academic environments.”*

*“I feel motivated to be part of a sports team in the University Athletic Association.”*

*“I believe my main motivation is to be enrolled in some extracurricular activities offered by my university.”*

### Negative emotions

Finally, the analysis revealed some responses associated with anxiety and demotivation, expressed as a desire to finish an undergraduate course as soon as possible, feeling relieved when thinking of it coming to an end and even not having any motivation to study medicine.

*“I just want to finish the undergraduate course as quickly as possible.”*

*“The course doesn’t motivate me.”*

## Discussion

According to SRL theory, learning is a process that is metacognitively guided, intrinsically motivated and strategic [1–3]. Recent studies have described SRL as an essential skill for medical students [45, 46], since the clinical workplace is complex and unpredictable. Additionally, as clinical knowledge is rapidly advancing, physicians need to continue learning autonomously, which also depends on SRL. Therefore, our study sought to investigate the relationship between metacognitive awareness and motivation to learn within the context of Brazilian undergraduate medical students.

We found that mastery approach-oriented students, whose priority is the development of competencies and skills, deeper learning and personal satisfaction, presented higher scores on one of the MAI domains (knowledge about cognition, *p* = 0.003, median 0.71, IQR 0.23). These findings corroborate the findings in the available literature about metacognition by reinforcing its relationship with better learning strategies, academic performance and academic success [9].

We also found that mastery avoidance-oriented students, characterized by avoiding the demonstration of weakness and academic difficulties, had lower scores on the domain of knowledge about cognition (*p* = 0.034, median 0.65, IQR 0.14). According to previous studies, avoidance-oriented goals are associated with poor psychological well-being, inadequate coping and learning strategies, procrastination, disorganization and lower grades [24–27, 43, 47, 48]. Therefore, our results also contribute to the literature by identifying maladaptive consequences of poorly developed SRL.

Knowledge about cognition refers to the interaction between person, task, and strategy [49]. It is related to self-knowledge and does not require specific training to develop [50]. On the other hand, regulation of cognition requires specific training as it is associated with learning skills [51]. Previous studies have already shown that students with higher mastery-oriented achievement scores have higher knowledge about cognition and no differences in the regulation of cognition [52]. However, there is no available evidence correlating the approach-avoidance categorization between the mastery goal orientation and the domains of metacognitive awareness.

Our findings address this gap in the differences between approach-avoidance categorization. It is important to consider that the score differences between knowledge about cognition and the two goal-orientation profiles are small, despite statistical significance. Therefore, we agree that this finding requires further investigation in different contexts, in order to confirm its reliability. Furthermore, additional studies should address whether encouraging mastery approach goals would enhance medical students’ metacognition.

It is known that the development of metacognition is directly associated with active learning methods such as flipped classrooms [53, 54], academic programmes such as mentoring [55] and constructive feedback [56]. There is also evidence that the educational environment can influence and change goal orientations, as long as teachers establish an open dialogue with students and help them identify mastery- and performance-oriented attitudes [55, 57]. Therefore, our findings represent an opportunity for faculty members to stimulate learning strategies and collaborative learning and provide a welcoming environment, as opposed to the current highly competitive environment, extensive workloads and abusive relationships in academic contexts [42, 56, 57].

According to previous studies, medical students’ motivation to learn is influenced by interests in science/medicine, social interests, altruism, flexible work hours, prestige, and financial security [34]. The present study, using qualitative analysis, allowed us to recognize some of the students’ motivational factors.

In our study, most of the students reported that they felt motivated to learn through case discussions and practical activities. This idea may be consistent with mastery goal orientations, since motivation focuses mostly on learning per se than on outcomes and feedback [24–26]. Although it could suggest an idea of enjoyable learning, it can also reflect insecurity and anxiety about not becoming a good doctor [57], consistent with the avoidance dimension.

Second, altruism, which concerns patient care and social responsibility, was an expected result, since humanistic values are one of the most prevalent reasons why people choose healthcare professions [32]. Students also reported being motivated by building a career and increasing their social status. Here, we can presume an association with performance orientation, since motivation is predominantly influenced by external rewards. Finally, since negative emotions were the least reported, further investigations of demotivation among medical students are needed.

Regarding medical education, Kusurkar states that motivation to learn is still underestimated in the construction of medical curricula [58]. The traditional curriculum is mostly influenced by the Flexner theory, focusing on the cognitive component of learning [59]. This model divides medical training into two different periods: basic sciences and clinical practice. It has been proven to be a partially ineffective model, since it can result in demotivation and a lack of interest among students [58]. This poorly integrated curriculum leads students to hardly understand the importance of the basic sciences for their future practice [59].

Current advances in medical training and curricular reforms already include problem-based learning (PBL), team-based learning (TBL), thinking aloud and mentoring [60, 61]. These active learning methods are student-centred and capable of promoting metacognitive awareness [31, 58]. However, there is still a lack of inclusion of methods focused on the motivation of students. Evidence states that motivation can arise from autonomy support, adequate feedback, and emotional support [58].

Our results can be used to make deans and faculty aware of the necessity of adopting strategies focused on promoting self-regulated learning concerning students’ motivational factors. We strongly believe that this mindset change is possible by applying the principles of self-regulation theory in medical education to enhance academic outcomes and promote enjoyable learning [5].

This study was designed to better understand a complex and multivariable picture: medical students’ potentialities and weaknesses in learning, thus encouraging an important discussion about the metacognitive, behavioural, motivational, and affective aspects of learning.

It is important to acknowledge institutional and cultural specificities that could have influenced the present results, for example, the predominance of mastery avoidance-oriented students (83%). Most studies until now have pointed to a mastery approach predominance of medical students’ goals [62]. However, none of them, for the best of our knowledge, were performed in Latin America. Besides, MAV goals are still underexplored and often omitted from most studies [63, 64].

The chosen cross-sectional design is adequate to investigate associations and provide wide-ranging data for discussion but does not allow for inferences of causality. In addition, since our sample was restricted to one medical school, further studies should investigate whether the trend cited above is replicable at other institutions. Therefore, we must be cautious with generalizations of these results to distinct populations.

## Conclusion

The majority of students endorsed a mastery avoidance goal orientation, which is significantly associated with lower metacognitive awareness. Students reported being motivated by aspects related to personal satisfaction, altruism and the medical curriculum. However, personal satisfaction was the aspect with the highest number of responses, while financial and social status had less importance. Understanding the metacognitive awareness and motivations of our students will help us to support them during medical training.

## Data Availability

The datasets used and/or analysed during the current study are available from the corresponding author upon reasonable request.

## Abbreviations

AGWD: Achievement Goal for a Work Domain
IQR: Interquartile range
MAI: Metacognitive Awareness Inventory
MAP: Mastery approach (goal orientation)
MAV: Mastery avoidance (goal orientation)
PAP: Performance approach (goal orientation)
PAV: Performance avoidance (goal orientation)
SRL: Self-regulated learning
USP: University of Sao Paulo
